# The Object Segmentation from the Microstructure of a FSW Dissimilar Weld

**DOI:** 10.3390/ma15031129

**Published:** 2022-01-31

**Authors:** Anna Wójcicka, Łukasz Walusiak, Krzysztof Mroczka, Joanna Krystyna Jaworek-Korjakowska, Krzysztof Oprzędkiewicz, Zygmunt Wrobel

**Affiliations:** 1 Department of Automatic Control and Robotics, AGH University of Science and Technology, 30-059 Cracow, Poland; jaworek@agh.edu.pl (J.K.J.-K.); kop@agh.edu.pl (K.O.); 2 Faculty of Materials Engineering and Physics, Cracow University of Technology, 31-864 Cracow, Poland; krzysztof.mroczka@pk.edu.pl; 3 Faculty of Architecture, Civil Engineering and Applied Arts, University of Technology, Rolna 43, 40-555 Katowice, Poland; lukasz.walusiak@wst.pl; 4 Institute of Biomedical Engineering, Faculty of Science and Technology, University of Silesia in Katowice, 41-205 Sosnowiec, Poland; zygmunt.wrobel@us.edu.pl

**Keywords:** FSW, image processing, computer vision, segmentation, microstructure analysis, dual-speed tool

## Abstract

Friction stir welding (FSW) is an environmentally friendly, solid-state welding technique. In this research work, we analyze the microstructure of a new type of FSW weld applying a two- stage framework based on image processing algorithms containing a segmentation step and microstructure analysis of objects occurring in different layers. A dual-speed tool as used to prepare the tested weld. In this paper, we present the segmentation method for recognizing areas containing particles forming bands in the microstructure of a dissimilar weld of aluminum alloys made by FSW technology. A digital analysis was performed on the images obtained using an Olympus GX51 light microscope. The image analysis process consisted of basic segmentation methods in conjunction with domain knowledge and object detection located in different layers of a weld using morphological operations and point transformations. These methods proved to be effective in the analysis of the microstructure images corrupted by noise. The segmentation parts as well as single objects were separated enough to analyze the distribution on different layers of the specimen and the variability of shape and size of the underlying microstructures, which was not possible without computer vision support.

## 1. Introduction

**Figure 1:**
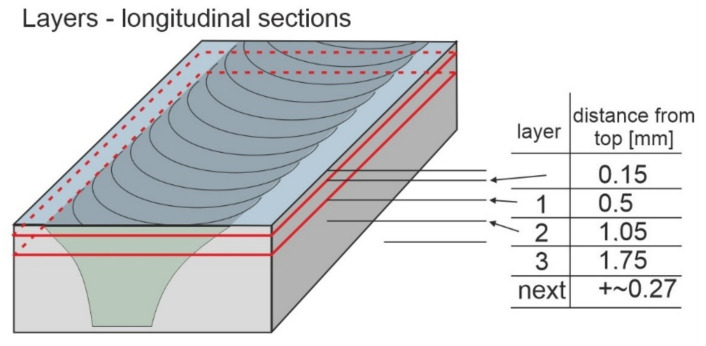
Multilayer weld metal and the scheme of performing longitudinal layers.

The friction stir welding (FSW) method, which was invented at The Welding Institute, Cambridge, UK, is a modern technology, enabling the welding and processing of various metallic materials [1,2,3,4]. FSW works by using a non-consumable tool to rotate and plunge into the interface of two workpieces. The tool is then moved through the interface, and the frictional heat causes the material to heat and soften (Figure 1). The basic idea of this method is a plastic deformation caused by the FSW tool to any material which remains in a solid state during the process [5]. The weld (also called welding joint) is a point or edge where two or more pieces of metal are joined together. The weld structure depends on welding parameters as well as types and configurations at the joint where actual welding can occur [6]. A zone within the weld where the tool performs the process is called the thermomechanical affected zone. There are multiple microstructure layers, which have been presented in Figure 1, which are created as an effect of mixing the materials and their plastic deformation to different extents, relevant to the space taken in the weld [7].

Furthermore, in the places that are significantly deformed, a recrystallization process occurs, which can be observed as a fragmentation of the microstructure (fine grains). If particles of a bigger size occur (about tens of microns) in the parent material, they are fractured [8]. As a result, those fragments can be subsequently distributed within the weld volume or moved to create precipitation bands. All these microstructure elements have a high impact on the mechanical properties of the weld and are, therefore, the subject of this research project. 

The novelty of this work can be summarized as follows:

- We present a new approach based on computer vision algorithms to segment layers occurring in the welding joint after applying the FSW method.
- We propose a methodology for object detection and analysis in multiple microstructure layers.
- The result of the application of a new type of the FSW tool (dual-speed) is also presented below.
- We compare and estimate the correlation between the feature importance and domain knowledge.

It is also important to stress that these microstructure elements occur in different FSW types and configurations at the joint where actual welding can occur.

This paper is organized into four sections as follows: Section 1 presents the motivation of this work and covers background information about the weld joint formation and microstructure occurrence in the deeper layers. Section 2 shows, in detail, the methodology used in this research to segment the weld joint sections as well as the microstructures with the use of computer vision algorithms. Section 3 presents the outcomes and a detailed analysis for a single weld joint, showing the disappearance of microstructures in deeper layers. Section 4 exposes the conclusions and suggests new lines of research.

### 1.1. Motivation

The analysis of the microstructure of engineering materials, welds in particular, is a challenging process that has to be supported by computer vision algorithms. Quantitatively analyzing the surface and shape of a given microstructure element (observed under the microscope) is normally extremely difficult as the analyzed object, or just its image, is composite itself. Encountering these cases required employing the method of manual object marking (putting a mask on them, briefly) or, in other words, attributing them with one common feature, which enabled the analysis. The advanced methods, especially different methods, and algorithms of digital image segmentation are of great importance for the material microstructure research [9]. In this paper, we describe a segmentation method based on thresholding in combination with domain knowledge, which allows this research using an FSW weld structure. The segmentation methods are based mainly on point image transformations. In most cases, these include various types of binarization, e.g., multi-criteria binarization with two thresholds [10,11,12,13], and also supportive processes of morphological image transformations [14,15,16], e.g., the processes of opening and closing [17,18,19]. A review of microstructural changes that occur during FSW in aluminum alloys and their modeling can be found in [20,21,22].

### 1.2. FSW Weld Images Specification

**Figure 2:**
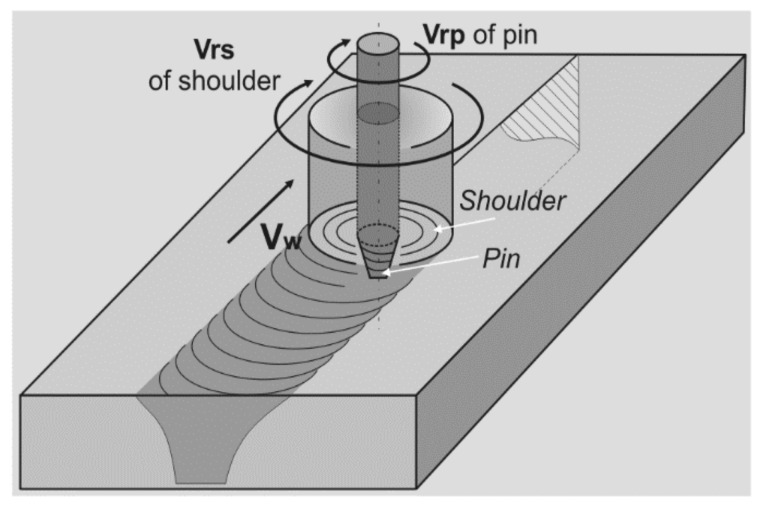
A schema of the FSW process with the dual-speed tool application.

In this research, we analyze the microstructure of FSW weld images of 2017A-T451 (in series production) and AlSi9Mg (EN AC-AlSi9Mg/3.2373 cast alloy) aluminum alloys. The weld was produced using a new experimental dual-speed FSW tool (Figure 2), with the following welding parameters: 1400 rpm—rate of a pin rotation, 350 rpm—rate of a shoulder rotation, and travel speed of 900 mm/min. Longitudinal sections have been prepared.

**Figure 3:**
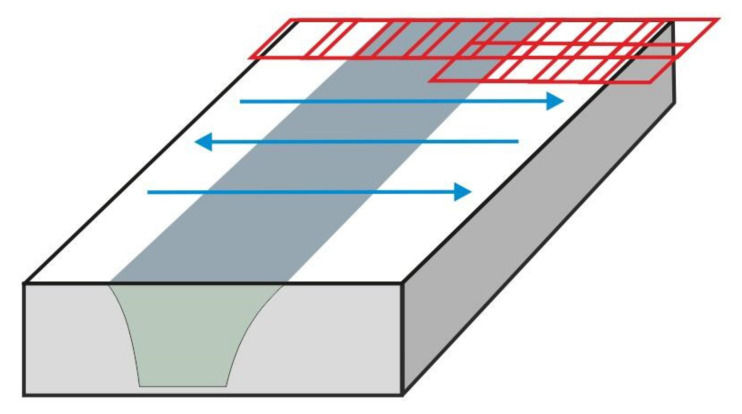
Methodology of taking a series of 64 photos by a light microscopy to create the macro-structure of the weld joint.

**Figure 4:**
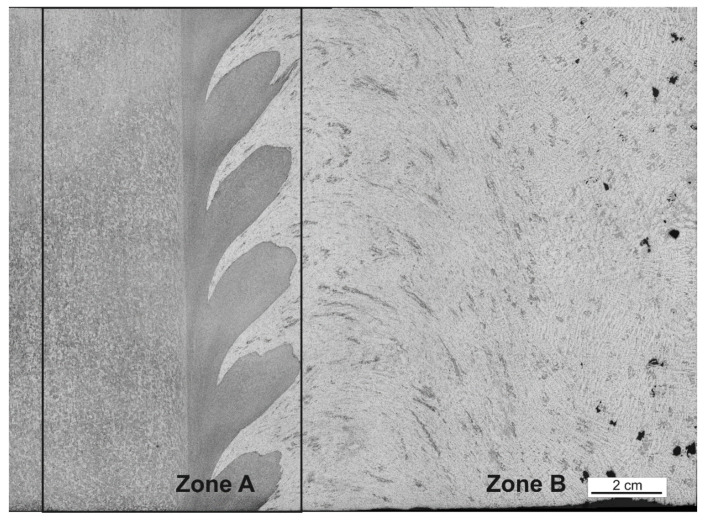
Division of the analyzed image into two zones: zone A represents the analyzed area, and zone B shows AlSi9Mg, the retreating side of the FSW weld.

**Figure 5:**
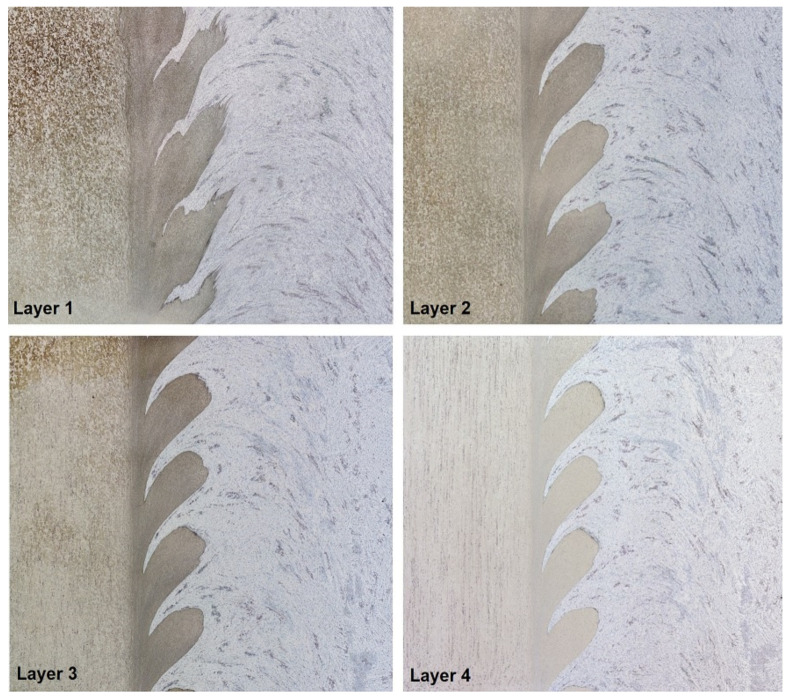
Visualization of four selected layers where the evolution of the microstructures can be observed.

The observed surfaces were polished mechanically and then etched with a solution of 2 mL HF, 4 mL HNO_3_, and 94 mL H_2_O. Macrostructures of the weld sections were created on the basis of microscopic images (magnification 50x) using an Olympus GX51 microscope. For testing purposes, for each of the seven layers, 64 images were set (connected) into the macrostructure, applying the Image Composite Editor (ICE) Microsoft software (Figure 3) where two clear zones can be distinguished (Figure 4. In Figure 5, we can observe the evolution of microstructures in four layers of the weld joint. Some of the microstructures disappear, which is a correct property; however, some still remain in the deep layers, which affects the properties of the weld and, therefore, should be analyzed.

For the purpose of the testing, we pointed out the A zone, which represents the analyzed area (processing zone), and the B zone, which is the retreating side of FSW performed on the AlSi9Mg material (Figure 5).

## 2. Weld Joint Analysis Framework

### 2.1. Image Preprocessing and Layers Segmentation

The image preprocessing stage, which is mostly obligatory before applying segmentation, is essential for the noisy images to remove the noise without substantially affecting the shape and number of objects that are present in the analyzed image. For this purpose, Matlab software was used.

As we can observe in Figure 2, the A zone is very homogeneous, and in order to perform the layer segmentation, we propose the following image processing steps: (1) image histogram equalization, (2) filtering, and (3) binarization. 

Histogram equalization is an image processing technique that adjusts the contrast of an image by using its histogram.

Formally, the histogram can be written as a function *h(l_k_),* defined as follows:

$$h\left(l_{k}\right)=\overset{M}{\underset{m=1}{\sum }}\overset{N}{\underset{n=1}{\sum }}p\left(l_{k},\left(m,n\right)\right)$$

where *h*(*l_k_*) is the sum of pixels with *l_k_* grayscale level and $\;p\left(l_{k},\left(m,n\right)\right)=\{1\;if\;L1\left(m,n\right)=l_{k}\;and\;0\;if\;L1\left(m,n\right)\neq l_{k}\;$}.

In this work, we used the global histogram equalization (GHE), which is a common technique used to enhance image contrast by utilizing the histogram information of the input image to create its transformation function. The process of histogram equalization can be presented as a shift in the position of consecutive columns containing information of a given grayscale level. A rule that governs this shift can be described as follows: some grayscale levels *L_a_* i *L_b_* belonging to the domain of function *h(l_k_) =* 0, every *l_a_< l_k_ < l_b_, L_a_* and *L_b_*_,_ have to be shifted to minimize the value coming from the formula:

$$Q=\left|\frac{\sum_{k=0}^{P}h\left(l_{k}\right)}{P}\right.-\frac{h\left(l_{k}\right)}{l_{a}-l_{b}}$$

For the purposes of the above histogram equalization, a given segment was transformed into a monochromatic image. Such an image is characteristic as it consists of grayscale. Presenting it on the graph would mean that values for the x axis range from 0 to 255 in grayscale, starting with black as 0 value, while the y axis mean number of pixels represents the existence of the given grayscale in the image.

**Figure 6:**
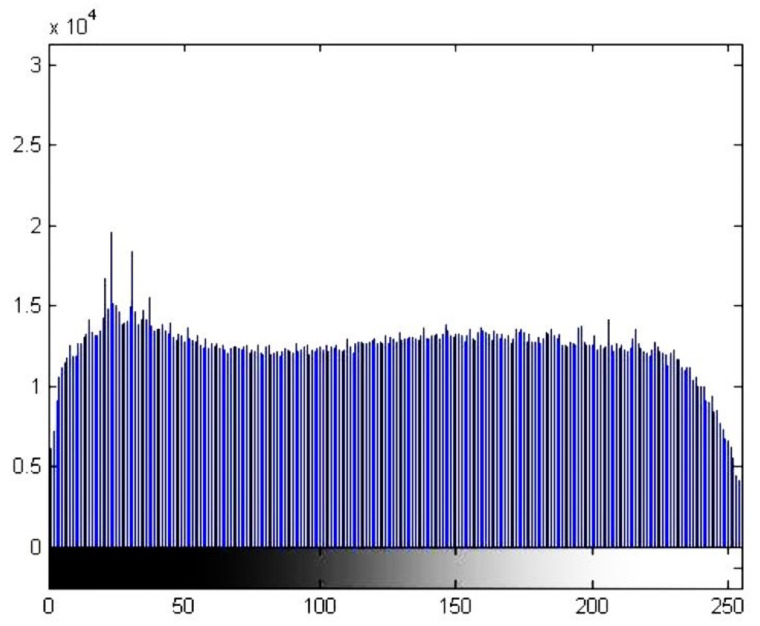
Histogram after performing global histogram adaptation and equalization using the exponential method for the image in Figure 2 (zone A).

GHE spreads out the most frequent pixel intensity values or stretches out the intensity range of the image, which can be observed in Figure 6.

**Figure 7:**
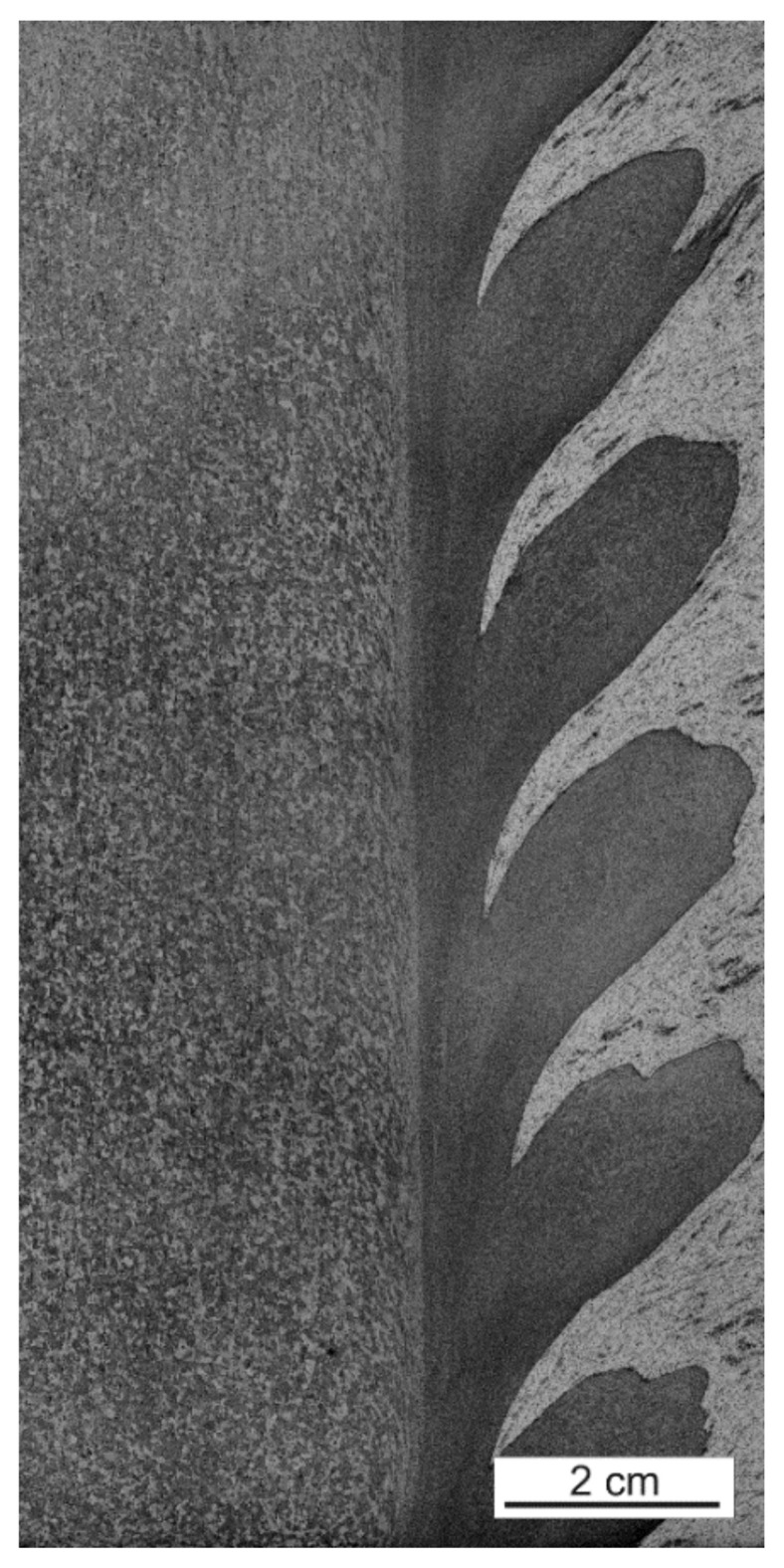
Zone A after the GHE histogram equalization. The differences between the surrounding and the welding layers can be observed.

**Figure 8:**
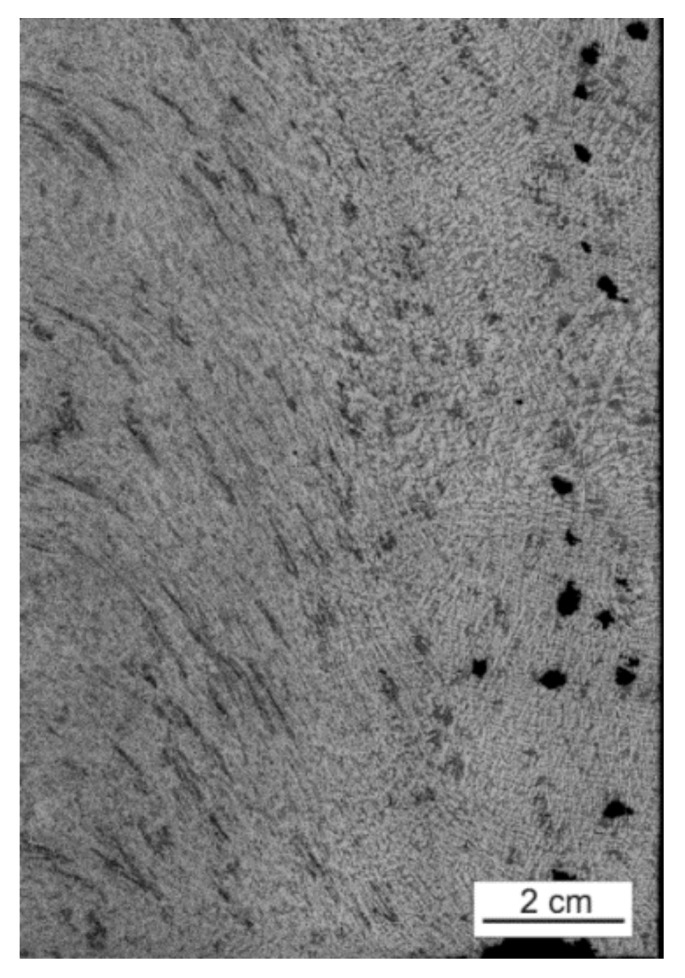
Part of the image after the histogram equalization and adaptation using the exponential method with the visible ‘grid’, which could not be clearly observed in Figure 2.

In Figure 7, we can observe the visible connections as well as detection of the border line present in the analyzed area of the casting alloy. The adaptation and histogram equalization with the exponential method were employed to obtain the visibility of microstructures (Figure 8).

A proper histogram equalization of the image with a high level of noise is a key action in the preliminary image preprocessing stage. It allows the highlighting of interested objects among the elements that comprise the noise layer. 

In the second step, a median filter [23], which is a non-linear technique, was applied to reduce higher noise densities present in the given image fragment. The median filter works by moving through the image pixel by pixel, replacing each value with the median value of neighboring pixels [24]. The median filter is useful as it is highly effective at removing noise while preserving edges in respective objects on the transformed image. 

The last step is the layer segmentation, which is performed using a multi-stage adaptive binarization scheme [25], consisting of two steps performed on the obtained images. This method is a combination of binarization with an upper threshold and binarization with a lower threshold. In the first step, the FSW weld image is separated into the background and layers based on an adaptive thresholding method. In the second stage, based on the outcome obtained in the previous step, the microstructure image is binarized using a global threshold, which is computed according to the histogram. This two-step binarization enables the obtainment of an even more precise result observed in the segmented structures. The operation can be described as follows:

$$L_{bin2PR}\left(i,j\right)=\left\{\begin{array}{c} 0\;for\;L\left(i,j\right)\leq p_{B1}\\ 1\;for\;p_{B1}<L\left(i,j\right)\leq p_{B2}\\ 0\;for\;L\left(i,j\right)>p_{B2} \end{array}\right.$$

where *p_B_*_1_, *p_B_*_2_ are the binarization thresholds, while required *p_B_*_1_ < *p_B_*_2_.

**Figure 9:**
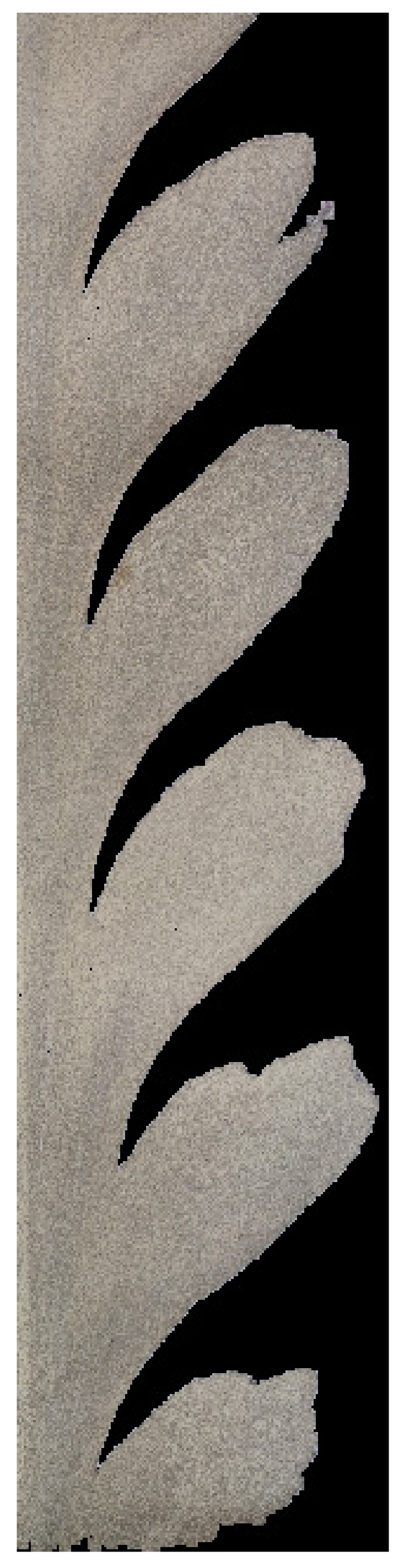
The FSW layer segmentation outcome based on the multi-stage adaptive binarization scheme.

Binarization thresholds values were defined after histogram analysis, whereas the histogram was obtained from the image already transformed using two of the steps mentioned above. In Figure 9, we present the outcome of the FSW welding joint layer segmentation.

Finally, the segmentation step is postprocessed by applying morphological operations, namely opening and closing. The goal of opening is to equalize the value of pixels for the objects to undergo the process of segmentation. There is one crucial rule to follow: after applying the first three steps of the given method, the objects cannot lose their properties and, even more importantly, their shape. After the opening process, the closing process is the next step. The opening process is a composition of accordingly performed processes of erosion and dilatation; it can be defined as follows:

$$C\left(L,SE\right)=L\;\cdot SE=E\left(D\left(L,-SE\right),-SE\right)$$

where *L* is the input image, *SE* refers to the structural element, and *E* and *D* are the morphological operations of erosion and dilatation, respectively.

The sequence of the applied processes is based on experimental studies.

Filling the openings on the resultative image is another step. This operation is performed to unify and embed objects after segmentation. After applying the consecutive steps, a wanted object that was supposed to obtain a full segmentation may encounter resultative inaccuracies in the form of objects with single missing pixels, which is a proven possibility. The function of this operation is to prevent unnecessary errors. Objects obtained in this way were tested for their area and location in the space of the given image, which was done to verify their location on the individual layers of microstructures.

### 2.2. Abrasions Detection in the Microstructure

**Figure 10:**
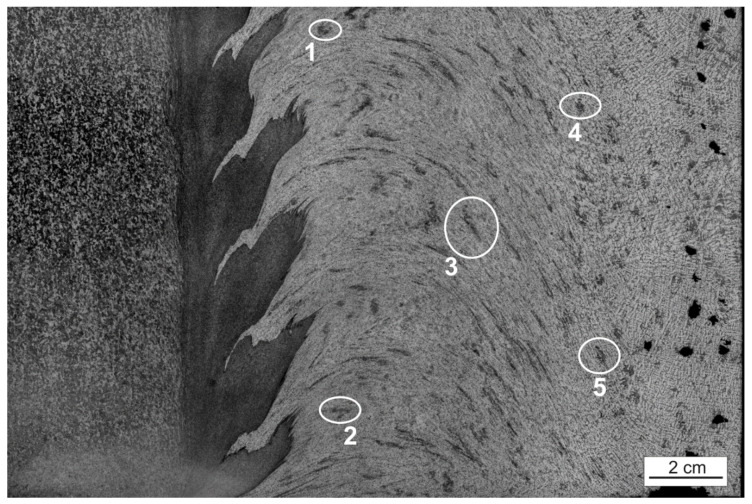
Highlighted specific objects (**1**–**5**) in the microstructure of the FSW welding joint layer.

Segmentation of the microstructures, which appear in the B zone, is the most important part in the analysis procedure of the weld joint and can be observed in Figure 10.

**Figure 11:**
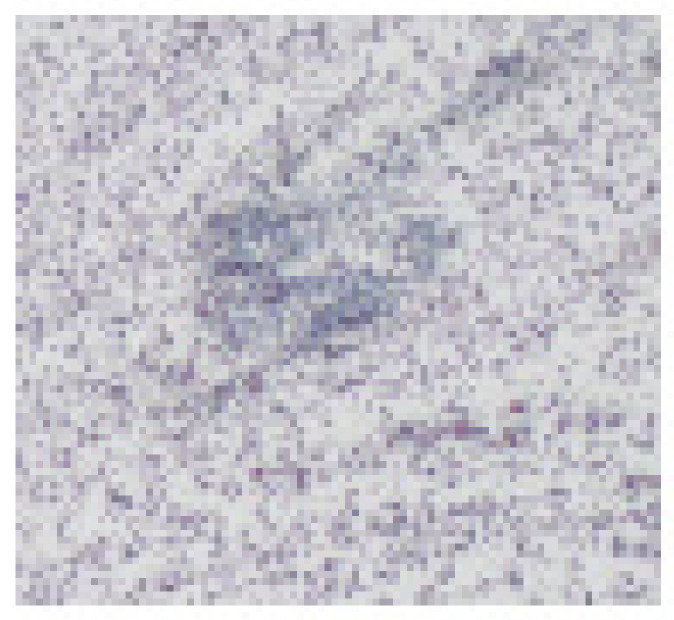
Part of the microstructure layer showing the object with the noise surrounding it. The object can be detected by a slightly different appearance and high density.

The purpose of this stage is to analyze how the objects change their position in relation to the main axes and their area in consecutive layers. Thanks to that, the directions and flux of the material as well as its mixing can be defined and determined. Five points of different properties and location in the space of B zone were picked on layer 1, and these points were enumerated to facilitate the interpretation of results, as shown in Figure 10. Afterwards, a segmentation of the objects was performed on each layer using the methods described in the previous step, including histogram equalization, median filtering, and binarization with histogram analysis. That was necessary due to the high intensity of image noise in this area, and without it, an exact definition of the object shape or even evaluation of its area are impossible, which can be observed in Figure 11. 

Subsequently, multiple trials were used to test and validate parameters in connection to the microstructure images. Finally, the parameters have been obtained experimentally [26,27,28].

## 3. Results

**Figure 12:**
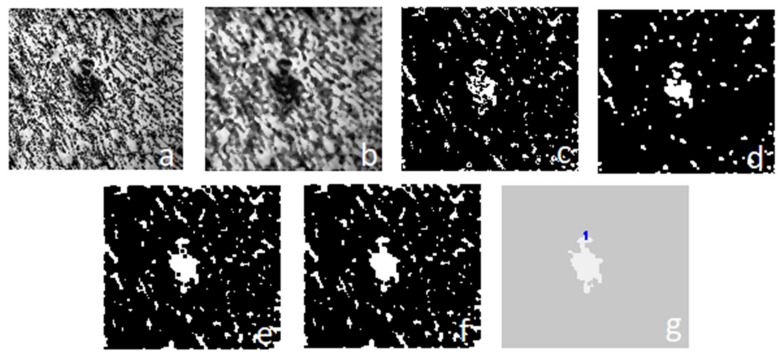
Stages of the object segmentation: (**a**) histogram adaptation and equalization; (**b**) median filter; (**c**) binarization using multi thresholding; (**d**–**f**) morphological operations, including opening and closing; (**g**) segmented area with the marked number.

**Figure 13:**
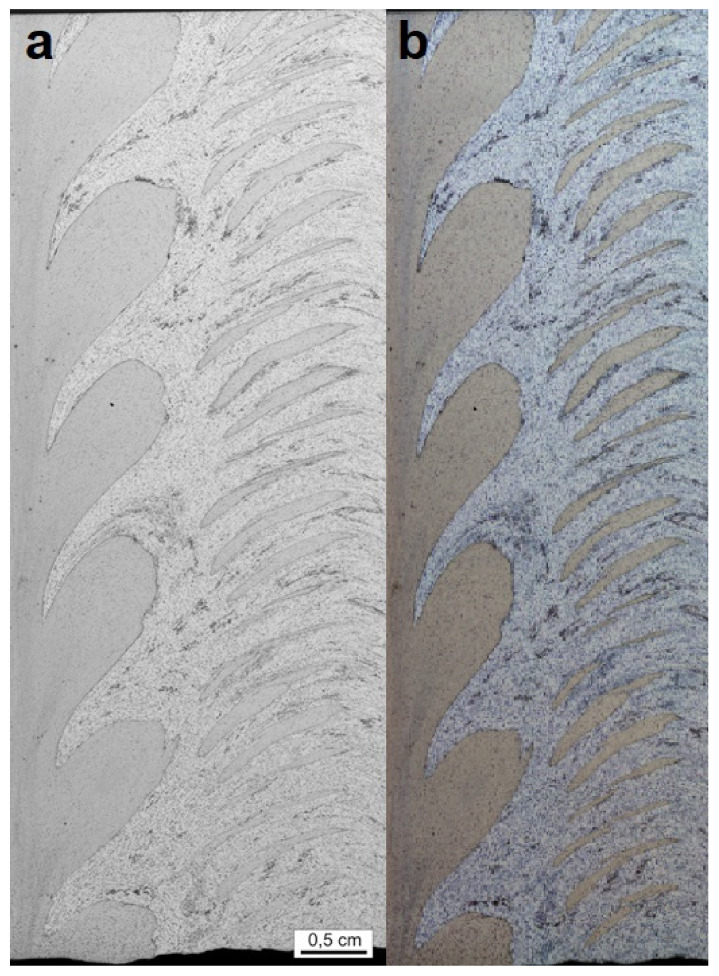
Abrasions visible in the microstructure: (**a**) an image without the square function transformation; (**b**) an image after the square function transformation.

After confirming that a border line between the layers and the surrounding area exists in the object (Figure 6), the thermomechanical affected zone with alloy 2017A underwent the process of segmentation, as shown in Figure 12. For that purpose, the focus was put on the color characteristic of the given fragment. Assuming that the wanted object color diagram is of the *K* value, a verification of the pixel value for a given object has been performed. Subsequently, it was assumed that if the *K* value range is known *<K_i_* … *K_j_ >*, all pixels within that range stayed in the unchanged form, whereas the rest were switched with 0 value (black). Non-linear image transformation, using the square function, was employed to enhance contrast in the layers 4–7 of the given macrostructure. This was performed due to the diverse quality of microstructure images and for the segmentation’s purpose. Applying the function mainly causes contrast enhancement in the area of the high-value pixels located in *m*,*n* for a given *L* image (Figure 13).

**Figure 14:**
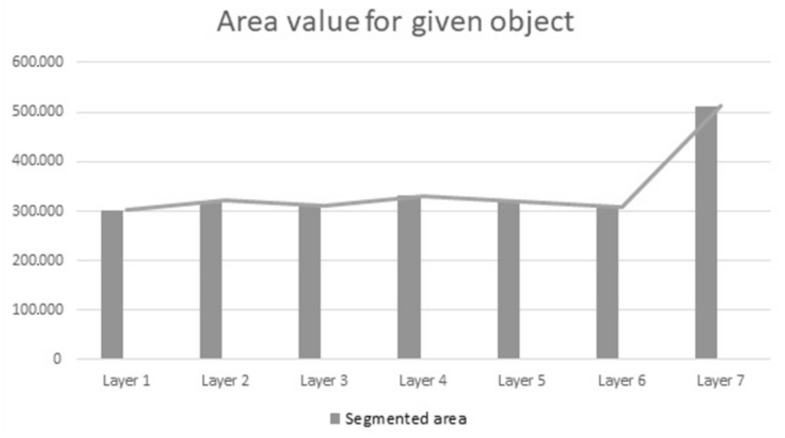
Visualization of the changes in the area during the progression of consecutive layers.

For each of the seven layers, a segmentation of a given area was conducted. At this stage, the way in which the area changes with the progression of consecutive layers was checked. Results are shown on the graph (Figure 14). It is clearly visible that layer 7 has the greatest change in comparison to all other layers. The reason behind this is that the specific area was added to objects (islands objects) created after removing another layer, and they merged with the main object. However, objects created in the process of uncovering another layer in B zone for layers 5 and 6 were not added; although they appeared, they didn’t merge with the main object.

An issue worth noting here is that on subsequent layers, it can be proven that in the thermomechanical affected zone, where the 2017A alloy (weld joint 2017A-T451, AlSi9Mg) is present, there is no difference in the area size of the analyzed fragment. It means, presumably, that the tested part of the microstructure has a similar structure between the layers and it does not change geometrically in the space defined by analyzed layers. On the other hand, there are new objects appearing on the microstructure, beginning with layer 5, with the characteristics as segmented objects. The square function for *L*(*m*,*n*) = (*L*(*m*,*n*))^2^ was employed (just as it was employed for segmentation of this object) to improve expected results. The only movable value for these images was pr value, a threshold of color intensity due to inadequacies within given microstructures.

For each of the objects, the center of the mass has been determined, and every consecutive layer was checked to view what object is present in the next layer with similar coordinates as in layer 1. The variability of objects and the extent of metal mixing in every layer were checked. The versatility of the function allowed it to work without the need to alter the values of structural elements (SE) in consecutive methods of this function, which clearly points to the fact that method selection order and their value are correct and versatile for this type of imaging of the FSW microstructures. The center of consecutive layers and the area of the given object were tested for every element. The results are shown in Table 1, which contains variability in the location of a given object center in relation to the coordinates. 

After an analysis of Table 2, it can be easily observed that in most coordinates values based on objects from layer 1 to 4, no objects occur or only a permanent layer of A zone occurs. Only selected coordinates values attributed to object 3 in subsequent layers indicate objects with various area values, but layers 5 to 7 indicate only single- or several-pixel objects and ‘island objects’, which appear to occur from layer 5. On the other hand, for object 5 in layer 5, an object with an area of 60 has been identified. 

To summarize the results in the table, the mixed layer is a layer of high variability. Objects present in the first layer no longer appear, even as small fragments in consecutive layers, and they are replaced with new objects, single pixels, or an extended layer of zone A. To make that possible, though, it is necessary to remove image noise in the zone of mixing. 

## 4. Conclusions

To summarize, we propose a methodology that accomplishes the following:

The devised method enables segmentations layers of a weld as well as objects occurring in the microstructures on images with a high level of image noise. Furthermore, there are many achieved outcomes.

It meets the criteria of efficient work and little computer performance consumption. 

It shows versatility for various layers of a microstructure, making the modifications during the tests less important. The application is useful in the process of transformation and analysis of the FSW microstructure.

Quantitative 3D analysis can successfully be a substitute, in this matter, for computed tomography [29]. 

The described two-stage framework for the analysis of the FSW weld joints reveals a viable application of the method to analyze the microstructure of heterogeneous materials. Thanks to computer vision algorithms, it is possible to describe objects in a given layer (including size and arrangement) but also determine their morphology (e.g., geometric changes) in a 3d system. Accuracy of this description will be dependent, of course, on how close the consecutive layers are located. Furthermore, in the spatial microstructure characteristic of the tested material, a description can be made regarding the intensity of occurrence of given particles (e.g., how their number changes from the area when getting deeper into the material). Such an application of the segmentation method brings up the association with 3d analysis and computed tomography, which can picture the structure of the analyzed preparation at large precision. However, it is important to consider that CT is efficient in cases where particle and structure density in tested material is significantly different. Other issues are availability of the device and substantial cost of the analysis. These limitations do not exist while applying the described method of object segmentation on the image of many layers of tested material. 

## Figures and Tables

**Table 1:** Location of a given object’s center in relation to the coordinate axis on each of the layers.

Object Type	Coordinates, such as Layer 1
Object 1	x824 y77
Object 2	x870 y1062
Object 3	x1206 y576
Object 4	x1490 y277
Object 5	x1548 y925

**Table 2:** Forming of the area of given objects on each of layers.

Coordinates:	Layer 1	Layer 2	Layer 3	Layer 4	Layer 5	Layer 6	Layer 7
Object 1	484	10	Single sets of pixels with values < 10	Object from A zone	Single sets of pixels with values < 10	Object from A zone	Object from A zone
Object 2	369	38	27	Object from A zone	Object from A zone	Object from A zone	Object from A zone
Object 3	941	489	35	872	Single pixels and border of abrasion in mixed layer	Single pixels and border of abrasion in mixed layer	Islands objects
Object 4	500	Single sets of pixels with values < 10	Single sets of pixels with values < 10	Single sets of pixels with values < 10	Single pixels and border of abrasion in mixed layer	48	51
Object 5	448	56	23	Single sets of pixels with values < 10	60	Single sets of pixels with values < 10	Single sets of pixels with values < 10

## Data Availability

All data is stored on AGH University of Science and Technology.
